# Therapeutic Effects of the Most Common Polyphenols Found in *Sorbus domestica* L. Fruits on Bone Health

**DOI:** 10.3390/nu18020267

**Published:** 2026-01-14

**Authors:** Noemi Penzes, Radoslav Omelka, Anna Sarocka, Roman Biro, Veronika Kovacova, Vladimira Mondockova, Monika Martiniakova

**Affiliations:** 1 Department of Botany and Genetics, Faculty of Natural Sciences and Informatics, Constantine the Philosopher University in Nitra, 94901 Nitra, Slovakia; noemi.penzes@ukf.sk (N.P.); asarocka@ukf.sk (A.S.); vmondockova@ukf.sk (V.M.); 2 Department of Zoology and Anthropology, Faculty of Natural Sciences and Informatics, Constantine the Philosopher University in Nitra, 94901 Nitra, Slovakia; roman.biro@ukf.sk (R.B.); vkovacova@ukf.sk (V.K.)

**Keywords:** *Sorbus domestica* L. fruits, bone health, chlorogenic acid, protocatechuic acid, rutin, epicatechin, naringin, osteoporosis, diabetes mellitus

## Abstract

The service tree (*Sorbus domestica* L.) fruits are rich in polyphenols, which exhibit promising therapeutic effects on bone health. This review summarizes the potential benefits of polyphenols identified in *Sorbus domestica* L. fruits, such as chlorogenic acid (CGA), protocatechuic acid (PCA), rutin, epicatechin, and naringin on bone biology and on bone-related diseases, including osteoporosis and diabetes mellitus. Current evidence suggests that the aforementioned polyphenols may modulate osteoblast and osteoclast activity, enhance mineralization, mitigate oxidative stress and inflammation, thereby supporting overall bone health. Specific studies highlight the anabolic and anti-resorptive effects of CGA, the osteoprotective potential of PCA, and the ability of rutin, epicatechin, and naringin to promote osteogenic differentiation and inhibit osteoclastogenesis. Although the exact mechanisms are still unclear, it is believed that these bioactive metabolites can act through a variety of signalling pathways and epigenetic mechanisms. Despite existing preclinical evidence, there is a significant gap in clinical trials evaluating the direct impact of polyphenols mentioned above on bone health in humans. Therefore, further research is needed to confirm their effectiveness in clinical settings. The therapeutic potential of the most common polyphenols from *Sorbus domestica* L. fruits has been evaluated by available in vitro and in vivo studies, which highlight their promising potential as dietary interventions to prevent bone loss and improve skeletal integrity in metabolic bone diseases. Based on available information, maximum health benefits may be achieved if mature *Sorbus domestica* L. fruits are consumed approximately two weeks after harvest or as unripe fruit-based fermented products.

## 1. Introduction

The service tree (*Sorbus domestica* L.) is a long-lived, typically 15–20 m tall tree (up to 30 m under favourable conditions) with imparipinnate leaves bearing 13–21 leaflets [1]. It is native to southern and central Europe, with notable presence in the Balkan Peninsula and Italy, and scattered populations in North Africa, the Caucasus, and Turkey [2]. The edible fruits (2–3 cm) resemble apples or pears and change from greenish-yellow to red-brown with sun exposure [3]. Both fruits and bark are used in traditional medicine to treat diarrhoea, vomiting, diabetes, and kidney stones due to their content of bioactive compounds with antioxidant, anti-inflammatory, and antidiabetic properties [4,5,6].

Osteoporosis and diabetes mellitus (especially type 2 diabetes mellitus-T2DM) are the most common metabolic bone diseases worldwide, and there is a complex pathological relationship between them. They often coexist, especially in the elderly, creating a “dual pandemic” in which metabolic disturbances compromise bone health (diabetic bone disease/secondary osteoporosis), leading to increased bone fragility and fracture risk [7,8,9]. Common risk factors such as unhealthy diet and physical inactivity exacerbate both of these conditions [10,11], while diabetes mellitus directly impairs bone cells and elevates fracture risk more than primary (postmenopausal and senile) osteoporosis [12], making bone health a crucial and often overlooked complication. In vivo models of diabetes mellitus have demonstrated elevated plasma levels of glucose, insulin (INS), total cholesterol, triglycerides, glycosylated haemoglobin (HbA1c) along with bone damage compared to non-diabetic subjects [13,14,15]. This finding confirms the systemic impact of chronic diabetes on both metabolic and skeletal health. 

In general, the aforementioned metabolic bone disorders are associated with increased production of reactive oxygen species (ROS) and inflammatory cytokines, such as tumor necrosis factor-alpha (TNF-α), interleukin-6 (IL-6), which, together with impaired glucose–INS signalling in diabetes, disrupt bone cell function and mineralization [16,17,18,19]. Diabetes mellitus and chronic inflammatory diseases often drive secondary skeletal dysfunction, reflecting the integrated role of redox biology, inflammation, and energy metabolism in bone health. Pharmacological agents, such as antidiabetic sulfphonylureas and anti-inflammatory glucocorticoids, can further elevate fracture risk and reduce bone mineral density (BMD) [20,21,22]. This underscores the potential of plant-derived polyphenols as complementary strategies to promote skeletal health in bone-related diseases. It is widely known that polyphenols have the ability to scavenge ROS, reduce chronic inflammation, lower hyperglycemia, increase INS sensitivity, improve INS secretion, and mitigate complications related to osteoporosis and diabetes mellitus [23,24,25]. 

*Sorbus domestica* L. fruits are rich in polyphenols, including chlorogenic acid (CGA), protocatechuic acid (PCA), rutin, epicatechin, naringin, and quercetin-3-β-glucoside (a major metabolite of quercetin) [26,27,28], which have been shown to modulate bone biology via direct and indirect mechanisms [29,30,31,32,33]. These include extracellular actions on bone cell receptors (e.g., estrogen receptors) and intracellular effects after absorption, modulation of signalling, gene expression, and metabolism [34]. Molecular docking calculations suggest that the aforementioned polyphenols may engage osteogenic mediators (such as TNF-α) and influence mineralization processes. Proposed mechanisms include direct binding to hydroxyapatite, interactions with collagen to enhance matrix properties, and modulation of epigenetic regulators, such as DNA methylation, histone modification, and non-coding RNAs [35,36]. While bone-specific data for many polyphenols remain limited, naringin, in particular, has shown protective effects mediated by miRNA pathways [37]. The bioactive outcomes may be modulated by plant matrix effects, protein–polyphenol interactions, and the generation of metabolites in vivo that differ from the parent compounds observed in vitro.

This review aimed to summarize the potential therapeutic effects of the most common polyphenols identified in *Sorbus domestica* L. fruits, including CGA, PCA, rutin, epicatechin, and naringin on bone biology and on bone-related diseases such as osteoporosis and diabetes mellitus by evaluating available in vitro and in vivo studies. It elucidates how these polyphenols may influence bone cell activity, mineralization, and overall bone integrity. Furthermore, it focuses on the physicochemical properties of the polyphenols and interactions with bone-related mediators and signalling mediators, as well as epigenetic modification of bone-related targets, and discusses their implications for the promotion of bone health and the prevention of metabolic bone diseases. Despite quercetin’s presence in *Sorbus domestica* L. fruits, we have excluded it from this review because its beneficial effect on bone health has been comprehensively described in our previous reviews [8,24,38]. However, quercetin was included in the chapter on polyphenol-polyphenol interactions.

## 2. *Sorbus domestica* L. Fruits: Nutrient Composition

There are limited data on the nutrient composition of *Sorbus domestica* L. fruits. A Bulgarian study examined pear-shaped fruits, while the study from Spain included two morphologically distinct fruit types, pear-shaped and apple-shaped. Overall, the Bulgarian samples were dominated by carbohydrates (approximately 14% of the fruit fresh weight-fw) with proteins around 1% fw and lipids around 0.25% fw [28]. In contrast, the fruits from Spain contained higher carbohydrate content (approximately 28–30% fw) and lower protein levels (0.54–0.68% fw) with lipids around 0.43–0.67% fw [39]. The macronutrient composition of *Sorbus domestica* L. fruits changes during maturation and postharvest ripening, although data are limited. An Italian study of *Sorbus domestica* L. fruits revealed that freshly harvested fruits had a higher starch content (approximately 267 mg/g fw), which decreased to approximately 48 mg/g fw during storage. Sugar content increased from 50 mg/g fw to 248 mg/g fw, suggesting a shift from complex to simple carbohydrates, which likely improves palatability. The protein profile changed during maturation, with enzymes associated with cell wall softening and sugar metabolism (e.g., β-galactosidase, malic enzyme) increasing, while enzymes related to starch synthesis decreased. Additionally, levels of certain lipid metabolites, including oleamide and hexadecanamide, varied, and terpenoid compounds generally decreased in later stages of maturation, which could have an impact on flavor and nutritional quality [40].

Ognyanov et al. [28] further characterized the fatty acid composition of *Sorbus domestica* L. fruits. Palmitic acid was the predominant saturated fatty acid, with linoleic acid being the main unsaturated fatty acid. Polyunsaturated fatty acid (PUFA) content was approximately twice that of saturated fatty acids, indicating a high-quality lipid fraction. β-sitosterol was identified as the most abundant sterol.

Regarding macroelements, Ognyanov et al. [28] reported lower total concentrations, with potassium (K: 155 mg/100 g fw) and phosphorus (P: 12.4 mg/100 g fw) being the most abundant. Calcium (Ca: 0.95 mg/100 g fw) was present in the fruits in smaller amounts. In contrast, Raigón Jiménez et al. [39] reported higher levels of K (185.5–221.2 mg/100 g fw), Ca (43.9–44.6 mg/100 g fw) and P (17.2–18.2 mg/100 g fw). In both aforementioned studies, the measured macroelements totaled less than 1% of the fw. Among the microelements, iron (Fe), copper (Cu), zinc (Zn), aluminum (Al), and boron (B) were represented in the samples [28,39]. Overall, *Sorbus domestica* L. fruits have a moderate mineral content compared to many other fruits, with the exception of vitamin B, which is notable for supporting bone health via calcium (Ca), vitamin D, and sex-hormone metabolism [41,42]. Seeds and bark showed higher Ca and magnesium (Mg) content compared to fruits [43]. Ca levels in seeds and bark were comparable to other Ca-rich foods such as cheese [44]. During fruit maturation, the levels of macroelements (e.g., Na, K, Ca) remained relatively stable [43].

Malic and quinic acids were the most abundant organic acids in the *Sorbus domestica* L. fruit organic-acid profile, with quinic acid acting as a key intermediate in the shikimate pathway. This compound may be involved in the biosynthesis of phenols (e.g., chlorogenic acids) in various species [28,45]. Ascorbic acid was qualitatively detectable but not quantifiable, with levels varying depending on genotype and extraction solvent [2,3,27].

The polyphenolic profile of *Sorbus domestica* L. fruits (including phenolic acids, flavonoids, and tannins) has been examined in several studies, with slight differences based on fruit maturity, geographical origin, and genotype [2,3,46]. The bark and immature exocarp exhibited the highest total polyphenol content, with the exocarp containing the greatest flavonoid content. Tannins were most abundant in the bark and mesocarp [43]. Well-ripened fruits, which are most commonly consumed, showed the lowest antioxidant capacity, while unripe fruits and the mesocarp (pulp) had higher capacity [47]. Unripe fruits are also rich in flavonoids [46]. For this reason, unripe fruit is processed into products such as vinegars to preserve high levels of polyphenols, antioxidants, and bioactive compounds [48]. In general, maturation tends to reduce the total polyphenol content in *Sorbus domestica* L. fruits, but the effects vary depending on the tissue (exocarp vs. mesocarp), maturity stage, and extraction method. A significant decrease in polyphenol concentration occurs within two to four weeks after harvest. However, the optimal period for consumption is approximately two weeks after harvest, when organoleptic properties improve and certain beneficial metabolites (e.g., citric acid) peak [40]. In this context, mature fruits are traditionally used for jams, jellies, compotes or alcoholic fermentation, which preserves a high level of beneficial compounds and enhances their palatability [27].

In general, the most abundant phenolic acids are CGA and its common forms 5-O-caffeoylquinic acid (5-CQA) and neochlorogenic acid (3-CQA), as well as protocatechuic acid (PCA, 3,4-dihydroxybenzoic acid). Among flavonoids, rutin, epicatechin, naringin, and quercetin are often detected [2,3,4,26,27,28]. 

Studies utilizing *Sorbus domestica* L. fruits are limited and focus primarily on their antioxidant, anti-inflammatory, and antidiabetic properties. Akkol et al. [4] demonstrated that extracts of *Sorbus domestica* L. fruits exhibited strong antioxidant and anti-inflammatory effects in a rat model of ulcerative colitis, as evidenced by elevated superoxide dismutase (SOD) and catalase (CAT) activities and decreased IL-6, TNF-α, caspase-3, myeloperoxidase (MPO), and nitrite in colonic tissue and blood. Hasbal et al. [5] reported that the plant extract potently inhibited α-glucosidase and elastase, with weaker effects on α-amylase, butyrylcholinesterase (BChE), and xanthine oxidase (XO). It indicates its promising potential in the treatment of diabetes mellitus and inflammation with possible neuroprotective implications. The antidiabetic activity of *Sorbus domestica* L. fruits may derive from inhibition of aldose reductase, which limits glucose-to-sorbitol conversion and mitigates osmotic and tissue damage under hyperglycaemic conditions [6,49].

In general, the antioxidant, anti-inflammatory, and antidiabetic properties of *Sorbus domestica* L. fruits suggest that their bioactive metabolites may modulate critical mechanisms (e.g., redox signalling, inflammatory responses, hyperglycaemia) that influence bone health.

## 3. The Most Common Polyphenols from *Sorbus domestica* L. Fruits: Their Effects on Bone Health

Research on the effects of *Sorbus domestica* L. fruits on bone health is currently lacking. Therefore, we selected polyphenols known to be present in these fruits, including CGA, PCA, rutin, epicatechin, and naringin, and conducted a literature search to investigate their impacts on bone biology and on bone-related diseases such as osteoporosis and diabetes mellitus. 

### 3.1. Chlorogenic Acid (CGA; 5-CQA)

CGAs include a family of caffeoylquinic acids that differ in the number and position of caffeoyl groups. 5-CQA is the most common form of CGAs identified in plants. It is an ester of caffeic and quinic acids found in coffee, stone fruits, apples, artichoke, Yerba Mate, and *Sorbus domestica* L. fruits [26,50]. *Sorbus domestica* L. fruits from Italy contained approximately 113.4 mg/100 g fw of CGA. In contrast, fruits collected from Turkey showed a higher CGA content, around 377 mg/100 g fw, both samples being collected at the mature stage [2,26]. Currently, CGAs often refer in particular to 5-CQA, which has been extensively studied due to its good availability in comparison with other isomers. CGAs are synthesized via the phenylpropanoid metabolic pathway, with three common and two restricted distinct biosynthetic pathways characterized in plants. In common pathways, L-phenylalanine produces trans-cinnamic acid, which is hydroxylated to trans-4-coumaric acid (p-coumaric acid, p-CA) and subsequently modified by downstream enzymes to form CGAs [51]. CGAs exhibit antioxidant, anti-inflammatory, and metabolic-modulating effects [52].

The bioavailability of 5-CQA involves sequential absorption and metabolism. About 33% of 5-CQA is absorbed intact in the upper gastrointestinal tract, approximately 7% in the small intestine after hydrolysis to caffeic and quinic acids, and roughly two-thirds reach the colon for microbial metabolism. The primary metabolites include p-CA, 3-(3-hydroxyphenyl) propionic acid (HPPA), and 3-(3-hydroxyphenyl) acetic acid (3-HPAA) [53,54]. Intact 5-CQA and metabolites enter the bloodstream and are further modified in the liver by methylation, sulfation, glucuronidation, glycine conjugation, hydrogenation, and dehydrogenation. Caffeic acid is converted to ferulic/isoferulic acids; quinic acid is transformed to gallic acid, then to p-hydroxybenzoic and syringic acids. In humans, 37 metabolites have been detected in blood, urine, and faeces, reflecting widespread biotransformation and excretion [54]. Most CGA is metabolized before reaching the systemic circulation, therefore its effects on bone in vivo are mediated by both the intact compound and metabolites. CGA has demonstrated potential anti-osteoporotic effects both in vitro and in vivo with anabolic stimulation of osteoblasts/osteoprogenitors, antiresorptive impacts on osteoclasts, and context-dependent inflammatory modulation. CGA at 0.1–10 μg/mL enhanced proliferation of MG-63 osteoblast-like cells, and reduced receptor activator of nuclear factor kappa-Β ligand (RANKL)-induced osteoclastogenesis in RAW264.7 macrophage cell line, indicating its dual anabolic/anti-resorptive actions [29]. In addition, treatment with 1 or 10 μM of CGA increased proliferation of bone mesenchymal stem cells (BMSC) and their differentiation to osteoblasts, with an important role of the Shp2/PI3K/Akt/cyclin D1 pathway [55]. CGA also promoted the nuclear factor erythroid 2-related factor transcription factor/Hemoxygenase 1 (Nrf2/HO-1) anti-oxidative pathway. In MC3T3-E1 preosteoblasts, dexamethasone (DEX)–induced overproduction of ROS was mitigated by CGA at 50 and 100 μM, which improved viability, activated Nrf2/HO1 axis, and downregulated Kelch-like ECH-associated protein 1 (Keap1). CGA also preserved p21 and reduced caspase-3/-9 activation, improving the B-cell lymphoma *2*/BCL2 associated X (BCL2/BAX) ratio and decreasing mitochondrial apoptosis [21]. Similarly, CGA reduced ROS and apoptosis in DEX-treated MLO-Y4 osteocytes (10, 100 μM) and upregulated bone-forming genes, including runt-related transcription factor 2 (*Runx2*), bone gamma-carboxyglutamate protein (*Bglap*, osteocalcin), β-catenin, *Cx43*. Mechanistically, CGA acted via HER2/AKT/mTOR signalling [56]. CGA in drug-containing serum (from rats treated with a 20 mg/kg of CGA) was able to increase the proliferation capabilities of osteoblasts and accelerate the transition process of G0/G1 phase to S phase, as well as enhance mitosis, regeneration of osteoblasts, and inhibit osteoblast apoptosis [57]. However, CGA can have pro-inflammatory effects in certain contexts. In TNF-α–driven MC3T3-E1 preosteoblasts, treatment with 50 μM CGA increased IL-6 release, indicating environment-dependent inflammatory modulation [58]. Thus, CGA may augment certain inflammatory outputs when inflammation is already present. Regarding the effect on osteoclasts, CGA (up to 50 μg/mL) inhibited osteoclast differentiation from mouse bone marrow-derived macrophages (BMMs) cultured with macrophage colony-stimulating factor (M-CSF) and RANKL [59]. Considering CGA metabolites, p-CA at concentrations of 1 and 4 μg/mL enhanced alkaline phosphatase (ALP) activity in the MC3T3-E1 cell line after 5 days of treatment, indicating a promotion of osteoblastic differentiation [60]. Similarly, various concentrations of HPPA (1, 5, 10, 50, and 100 µg/dL) applied to ST2 cells (mouse bone marrow-derived stromal cells) for 10 days demonstrated increased osteoblastic differentiation. Furthermore, mRNA levels of collagen type I alpha 1 (*COL1A1*) and osteoprotegerin (*OPG*) were elevated in the 5 and 50 µg/dL dose groups following 24 h of treatment [61]. On the other hand, an application of HPPA at concentrations of 1, 10, and 100 µg/dL to RANKL-induced RAW 264.7 cells resulted in a significant reduction in osteoclast differentiation [62].

In ovariectomized (OVX) rats, 50 and 75 mg/day of CGA for 8 weeks significantly improved trabecular number (Tb.N), trabecular thickness (Tb.Th), bone volume fraction (BV/TV), and mechanical properties of the tibia [29]. In another study, 27 and 45 mg/kg/day of CGA for 12 weeks attenuated estrogen deficiency–induced bone loss in rats, preserving BMD and trabecular bone microarchitecture, and increasing serum osteocalcin and ALP levels [55]. In addition, CGA at 100 mg/kg/day for 4 weeks improved skeletal health in female rats regardless of estrogen status [63]. Complementing these findings, CGA at 45 mg/kg/day for 12 weeks in OVX rats significantly enhanced bone microarchitecture of the femur [64]. Finally, CGA protected bone integrity in rat models of femoral head necrosis by reducing inflammation and infection. At a dose of 20 mg/kg/day for 4 weeks, it increased femoral head BMD, improved blood viscosity, and boosted osteoblast proliferation, while lowering BAX and raising BCL2 to reduce apoptosis [57]. The beneficial effects of CGA on bone health have also been obtained using mouse models. Supplementation with 0.5 mg/kg/day of CGA for 4 weeks improved bone microstructure and increased ALP and procollagen type 1 *N*-terminal propeptide (P1NP) levels in DEX-induced osteoporotic mice [56]. In addition, CGA (10 mg/kg/day for 8 days) reduced lipopolysaccharide (LPS)-induced bone erosion and improved trabecular bone microarchitecture [59]. Regarding CGA metabolites, p-CA (100 mg/kg for 10 days) facilitated longitudinal bone growth in adolescent rats by stimulating chondrocyte proliferation and upregulating insulin-like growth factor 1 (IGF-1) expression [65]. In OVX rats, the administration of p-CA at a dosage of 10 mg/kg/day for 4 weeks resulted in a significant enhancement of bone mass [66]. Application of HPPA to healthy mice at concentrations of 0.5 and 1 mg/kg/day for 30 days significantly improved bone microarchitecture (higher BMD, BV/TV, Conn. D, and Tb.Th). Additionally, an increase in bone turnover markers, including ALP and osteocalcin, was determined [61].

To date, no clinical trials have directly determined CGA’s effects on bone health. Nonetheless, trials using green coffee bean polyphenols (rich in CGA isomers) showed certain metabolic benefits. In a metabolic syndrome study (body mass index-BMI > 25 kg/m^2^), decaffeinated green coffee bean extract (400 mg twice daily for 8 weeks) significantly reduced fasting blood glucose (FBG) level, improved INS resistance (HOMA-IR), and decreased body weight [67]. A novel formulation derived from green coffee bean extract, GCB70^®^ (70% 5-CQA, 30% 3-CQA; <1% caffeine) administered to overweight participants at 500 mg twice daily for 12 weeks, significantly reduced plasma levels of leptin, FBG, HbA1c, thyroid-stimulating hormone (TSH), and low-density lipoprotein (LDL). On the other hand, high-density lipoprotein (HDL) levels were elevated [68]. In another randomized trial, participants consumed polyphenol-rich lettuce cultivated under saline eustress and high in CGA (100 g/day for 12 days). Improvements in total cholesterol, LDL cholesterol, vitamin D and P levels were recorded compared with the control group receiving standard lettuce. However, no significant differences in bone turnover markers (osteocalcin, C-terminal telopeptide of type 1 collagen-CTx) were found, likely due to the short duration [69]. Collectively, aforementioned clinical trials indicate certain metabolic benefits from green coffee bean polyphenols containing CGA isomers that could plausibly influence bone health, but direct bone-specific evidence remains lacking.

In summary, the preclinical evidence supports bone-protective effects of CGA, as summarized in Table 1.

### 3.2. Protocatechuic Acid (PCA)

PCA is a hydroxybenzoic acid found in bran, brown rice, grapes, chicory, star anise, and *Sorbus domestica* L. fruits [28,70,71]. It occurs free or conjugated, produced via the shikimate/phenylpropanoid pathways. The PCA content was approximately 886 mg/100 g dry weight (dw) in mature *Sorbus domestica* L. fruits collected in Bulgaria [28], while in fruits from Bosnia and Herzegovina it was approximately 5.1 mg/100 g g dw [27]. In general, PCA exhibits antioxidant, anti-hyperglycaemic, anti-inflammatory, antibacterial, antitumor, and neuroprotective activities [72].

PCA has limited intact bioavailability. After oral dosing, PCA is mainly absorbed in the small intestine and rapidly metabolized in the liver to sulphate and glucuronide conjugates (e.g., PCA-3-sulfate, PCA-4-sulfate, PCA-glucuronide). The kidney is a major excretory route [73,74]. In humans, chicory-derived PCA is found as both the parent compound and its metabolites, with reported distribution of approximately 24% in blood, 12% in urine, and 13% in faeces. The exact proportion of intact PCA remains uncertain [71]. Consequently, bone effects are likely driven by PCA metabolites or local biotransformation rather than the unmetabolized compound, which may explain discrepancies between in vitro and in vivo findings. 

PCA has demonstrated beneficial effects on bone health in in vitro and in vivo animal model studies. In primary human osteoblast cultures, PCA at 50 and 100 μM increased the expression of osteogenic genes *SPP1* (osteopontin), integrin-binding sialoprotein (*IBSP*), tissue non-specific alkaline phosphatase (*ALPL*), and bone morphogenetic protein 6 (*BMP6*). PCA also induced the expression of the anti-osteogenic marker interleukin-1 beta (IL-1β). However, statistical analyses were not conducted to confirm significance [75]. Overall, PCA improved bone health by mitigating glucocorticoid-induced osteoblast dysfunction. At 12.5, 25, and 50 μmol/L, PCA enhanced proliferation in DEX-treated MC3T3-E1 preosteoblasts. Additionally, PCA enhanced osteoblast differentiation, indicated by increased ALP levels, reduced ROS and malondialdehyde (MDA), and boosted antioxidant enzymes SOD and glutathione (GSH) [76]. PCA application at 8 μM reduced RANKL-induced tartrate-resistant acid phosphatase (TRAP) production in the RAW264.7 cell line, inhibiting osteoclast differentiation. This treatment also decreased ROS levels, increased antioxidant enzymes (SOD, glutathione peroxidase-GPx, GSH), and downregulated osteoclast markers such as *Trap*, TNF receptor-associated factor-6 (*Traf6*), proto-oncogene tyrosine-protein kinase Src (*c-Src*), cathepsin, matrix metalloproteinase-9 (*Mmp9*), nuclear factor of activated T-cells (*Nfatc1*), and activator protein 1 (AP-1). PCA inhibited mitogen-activated protein kinase (MAPK) and inflammatory pathways and enhanced apoptosis by increasing mitochondrial membrane potential and caspase activity [77]. In another study using the same model and conditions, 8 μM of PCA similarly suppressed osteoclastogenesis, reducing TRAP-positive osteoclasts [78]. PCA treatment at 80 μM of murine C3H10T1/2 mesenchymal stem cells (MSCs) significantly increased mineralization, Ca/P deposits, and ALP activity. It upregulated osteogenic genes *Runx2*, *Alpl*, and osterix (*Sp7*) while suppressing adipogenesis [79]. 

In OVX mice, PCA treatment (10 and 20 mg/kg/day for 12 weeks) improved trabecular bone microarchitecture (higher BV/TV, Tb.N, Tb.Th) and lowered RANKL while raising OPG (at 20 mg/kg/day). Additionally, PCA administration reduced expression of key osteoclast-related genes in bone marrow–derived cells, including *Traf6*, *Nfatc1*, and the calcitonin receptor [31]. Furthermore, PCA administration (50 mg/kg for 40 days) in a murine model of alcohol-induced bone loss significantly improved trabecular bone parameters of the tibia, including BMD, BV/TV, and Tb.N [80]. 

To date, no clinical trials have evaluated the effects of PCA or its derivatives on bone health, either directly or via predefined biomarkers. Since PCA is a major metabolite of anthocyanins in vivo, the broader literature on anthocyanins offers indirect context. Anthocyanins occur in *Sorbus domestica* L. fruits and have demonstrated metabolic benefits (improved lipid profiles, lower FBG, and increased antioxidant capacity) that could plausibly affect bone health in metabolic syndrome conditions, though direct effects are not known [2,39,81,82]. Well-designed human studies are needed to clarify direct or indirect bone-specific effects of PCA or anthocyanin-derived PCA.

The aforementioned data indicate that PCA exhibits a multifaceted bone-protective effects in vivo and in vitro, promoting osteoblast activity and mineralization while inhibiting osteoclast differentiation and activity, with antioxidant and anti-inflammatory mechanisms likely contributing to its overall bone-sparing action (Table 2).

### 3.3. Rutin (Quercetin-3-O-Rutinoside)

Rutin is a flavonol glycoside of quercetin linked to rutinose. It is widespread in plants, especially in buckwheat, tea, citrus, and *Sorbus domestica* L. fruits [3,83]. The rutin content in mature *Sorbus domestica* L. fruits collected in Turkey was 125 mg/100 g fw in one study, while another study reported a higher value of 600 mg/100 g fw [2,3]. Rutin has limited water solubility, with greater solubility in organic solvents such as alcohols, influencing its stability, bioavailability, and activity [84]. Pharmacologically, rutin has antioxidant, anti-inflammatory, vasoprotective, and neuroprotective properties via modulation of oxidative stress and inflammatory pathways [85].

Rutin has low oral bioavailability (20%), driven by its size, glycoside form, and poor solubility [86,87]. It largely reaches the colon unmetabolized, where gut microbes hydrolyse it to quercetin and short-chain phenolics (e.g., 3,4-dihydroxyphenylacetic acid, homovanillic acid) [88]. Phase II enzymes conjugate rutin and quercetin to sulfates or glucuronides. These conjugates can circulate and be deconjugated at target tissues (e.g., liver, kidney, lungs, brain) to exert their effects [85,89]. Bioavailability can be enhanced through encapsulation (e.g., nanoparticles, phospholipid complexes) [90].

Rutin exhibits multilevel effects on bone biology, with evidence from in vitro, in vivo animal model studies, and limited human data. In an in vitro study, rutin at 1 μM enhanced osteogenic proliferation and differentiation in human periodontal ligament stem cells (PDLSCs). It also upregulated COL1A1, ALP, RUNX2, and SPP1 (osteopontin) at both mRNA and protein levels [91]. In human osteoblast-like MG-63 cells, rutin application (10 and 25 μg/mL) enhanced osteogenic differentiation by boosting proliferation, ALP activity, collagen formation, and mineral deposition [92]. Another study demonstrated that rutin (50 mg/mL) induced osteogenic differentiation of MSCs over adipocyte formation, mediated by the downregulation of p53, upregulation of extracellular matrix components, and activation of osteogenic genes such as *ALPL*, *BGLAP*, *SPP1*, and *RUNX2* [93]. Isolated mouse bone marrow cells incubated with M-CSF and RANKL and treated with rutin (20 μM) showed a significant reduction in osteoclast numbers, ROS, and TNF-α levels, which was associated with inhibition of NF-κB signalling [94].

Rutin demonstrated therapeutic potential in osteoporosis treatment based on in vivo data. In an OVX mice model, intraperitoneal administration of rutin (50 mg/kg/day for 4 or 8 weeks) improved lumbar vertebrae and femoral bone histomorphometry (increased BV/TV, Tb.Th), and lowered inflammation (reduced IL-1β, IL-6, and TNF-α). Additionally, serum ALP and CTx levels were also decreased compared with the OVX group, indicating lower bone resorption [32]. Similarly, supplementation with rutin (10 mg/kg/day for 3 months) in OVX rats increased BMD and reduced levels of IL-6, TNF-α, and interferon-gamma (IFN-γ) [95]. Rutin (2.5 mg mixed with 900 mg of demineralized freeze-dried bone allograft) has been shown to accelerate bone repair in a rabbit model over 6 weeks, potentially by decreasing the gene expression of matrix metalloproteinases (MMPs) and increasing the expression of collagen type III (*COL3A1*) [96].

There is limited evidence for the direct application of rutin in humans and for monitoring its effects on the skeletal system. However, rutin application (500 mg/day for 3 months) in a randomized, double-blind, placebo-controlled trial improved glycaemic control in patients with T2DM, as indicated by lower FBG, INS, HbA1c, and HOMA-IR, as well as improved INS sensitivity and pancreatic β-cell function. It also enhanced lipid profiles and provided antioxidant/anti-inflammatory benefits (higher total antioxidant capacity, lower IL-6 and MDA levels) [97]. In another clinical trial, the impact of rutin (1 g/day; 500 mg of pure rutin combined with other components for 3 months) in patients with T2DM was investigated. Rutin ameliorated blood pressure and elevated activities of SOD and GPx [98]. 

Based on these findings, rutin exhibits osteogenic effects in animal model studies and in vitro experiments, as well as anti-osteoclastogenic potential via NF-κB inhibition (Table 3).

### 3.4. Epicatechin

Epicatechin is a flavanol (aglycone dominant, with glycosides such as epicatechin-3-O-glycosides) found in cocoa, dark chocolate, green tea, apples, grapes, and *Sorbus domestica* L. fruits [3,99]. The epicatechin content in *Sorbus domestica* L. fruits from Turkey was approximately 293.5 mg/100 g fw. In contrast, ripe fruits from Bulgaria contained about 22.2 mg/100 g fw of epicatechin [3,28]. The flavan-3-ol structure confers strong antioxidant activity via multiple hydroxyl groups. Epicatechin demonstrates poor water solubility but solubility in alcohols. The aglycone epicatechin is more lipophilic and better absorbed than glycosides, which gut microbiota can hydrolyse to the aglycone [100,101]. Pharmacologically, epicatechin exhibits antioxidant, anti-inflammatory, vasodilatory, INS-sensitizing, neuroprotective, and osteo-modulatory effects, acting through free-radical scavenging and modulation of nitric oxide (NO) signalling and mitochondrial function [102]. These properties support its potential for improving skeletal health [103]. 

Epicatechin has moderate oral bioavailability from partial absorption and extensive first-pass metabolism. Absorption occurs mainly in the small intestine; aglycones are absorbed better than glycosides. It undergoes glucuronidation, sulfation, and methylation to conjugated metabolites such as epicatechin-3′-O-glucuronide, epicatechin-3′-O-sulfate, and 3′-O-methyl-epicatechin, which dominate in plasma and reach liver, kidney, brain, and vasculature. Unabsorbed epicatechin is metabolized by gut microbiota to phenolic acids (e.g., 5-(hydroxyphenyl)-γ-valerolactones) with systemic activity. Advanced strategies like nanoparticle formulations and bioenhancers aim to improve stability and transport [104,105,106]. 

Epicatechin and its derivatives show significant osteoprotective potential in preclinical studies; however, evidence of their positive effects on bone health in humans remains limited. Treatment of human MSC with 1 or 100 µM epicatechin in combination with diluted osteogenic medium significantly upregulated osteogenic marker *RUNX2* and increased secreted protein acidic and cysteine rich (*SPARC*; bone-linked matricellular protein) expression [107]. Additionally, epicatechin significantly enhanced rat osteoblast proliferation at 25 and 50 μg/mL, as monitored by enhanced ALP activity, mineralization, and hydroxyapatite formation. Furthermore, epicatechin demonstrated a significant protective effects against hydrogen peroxide-induced cell damage in C2C12 mouse myoblasts, highlighting its osteoprotective properties through antioxidant mechanisms [108]. Finally, treatment with epicatechin 3-O-β-D-allopyranoside (ECAP, a glycosylated epicatechin derivative) at 50 and 100 μg/mL reduced TRAP-positive osteoclasts, probably by downregulating RANKL-induced NF-κB and NFATc1 signalling [30] (Table 4).

In vivo, epicatechin exhibited substantial osteoprotective properties by stimulating bone formation and reducing bone resorption, making it a compelling candidate for managing osteoporosis. Supplementation with ECAP at 100 mg/kg/day for 4 weeks mitigated OVX-induced bone loss in mice, which was manifested by improved trabecular bone microarchitecture [30]. 

There are no clinical studies investigating the impact of epicatechin on bone health. However, in a large study involving 21,442 healthy participants, the daily cocoa extract supplement containing 500 mg/day flavanols and 80 mg/day epicatechin was not associated with lower risk of incident clinical fracture [109]. Nevertheless, epicatechin supplementation can improve INS sensitivity and postprandial metabolism in humans. In a 4-week long, randomized, double-blind, placebo-controlled crossover study in adults with elevated systolic blood pressure, daily epicatechin administration (100 mg) improved fasting INS and HOMA-IR, whereas quercetin did not, suggesting that epicatechin contributes to the cardiometabolic benefits of flavonoid-rich foods [110]. Similarly, in postprandial tests, oral epicatechin at 1 mg/kg increased lipid oxidation and lowered plasma glucose and triglyceride (TAG) levels with greater benefits in overweight adults [111]. In postmenopausal women with T2DM, one year of flavonoid-enriched chocolate (90 mg epicatechin daily, combined with flavan-3-ols and isoflavones) improved HOMA-IR, INS, and lipid levels, and reduced estimated 10-year coronary heart disease risk without altering HbA1c or blood pressure [112]. Even absent direct evidence that epicatechin alters bone parameters, its favourable impacts on INS sensitivity and glucose and lipid metabolism may indirectly benefit bone health. 

### 3.5. Naringin

Naringin (naringenin-7-O-neohesperidoside) is a major citrus flavanone glycoside with low oral bioavailability (≈5–9%) due to poor solubility and glycosylation. In ripe *Sorbus domestica* L. fruits from Bulgaria, naringin constituted 16 mg/100 g fw [28]. After ingestion, intestinal α-rhamnosidase and β-glucosidase rapidly deglycosylate it to naringenin, the more absorbable aglycone, which then undergoes extensive phase II metabolism [113,114]. 

The pharmacokinetics of naringin vary across species. Bioavailability is 44.1% in rats and 34.4% in dogs, reflecting substantial first-pass metabolism. In humans, naringin shows a longer half-life with extensive metabolism; about 12 metabolites have been identified [115]. Gut microbiota generate a broad range of naringin catabolites (methylated and hydroxylated phenolics) and metabolites such as neoeriocitrin, rhoifolin, and naringenin, which contribute to bioactivity in vivo [116]. Together with hepatic conjugation and cytochrome P450 (CYP)-mediated metabolism, this highlights naringin’s complex pharmacokinetic profile. To improve clinical efficacy, advanced formulations or prodrug strategies may be needed to enhance bioavailability.

In MC3T3-E1 preosteoblasts, naringin (0.5–1 μM) increased proliferation and ALP activity, enhanced mineralization, and upregulated RUNX2, COL1A1, and osteopontin at the gene and protein levels, indicating stimulated osteoblast differentiation. Supplementation with naringin (30 mg/kg/day for 12 weeks) improved trabecular bone microarchitecture in OVX mice (increased BMD, BV/TV, Tb.N, Tb.Th; decreased Tb.Sp) and elevated serum P1NP and osteocalcin levels, while lowering CTx [117]. A comprehensive review by Nor Muhamad et al. [33] documented the positive effects of naringenin on the skeletal system through several key signalling pathways, including BMP-2/p38MAPK/RUNX2/Osx, SDF-1/CXCR4, and PI3K/Akt/c-Fos/c-Jun/AP-1 [33] (Table 5).

There is limited clinical evidence on the effects of naringin on the human skeleton. A small pilot study in a single female subject with diabetes found that oral naringenin, the aglycone (sugar-free) version of naringin (150 mg three times daily for 8 weeks), reduced body weight, increased resting metabolic rate, and lowered INS levels without significant influence on glucose [118]. In primary human adipocytes, naringenin increased oxygen consumption and upregulated uncoupling protein 1 (*UCP1*), glucose transporter type 4 (*GLUT4*), and carnitine palmitoyltransferase 1 beta (*CPT1β*) via peroxisome proliferator-activated receptor alpha (*PPARα*) and gamma (*PPARγ*) signaling, suggesting its potential to boost energy expenditure and improve insulin sensitivity [118].

**Figure 1:**
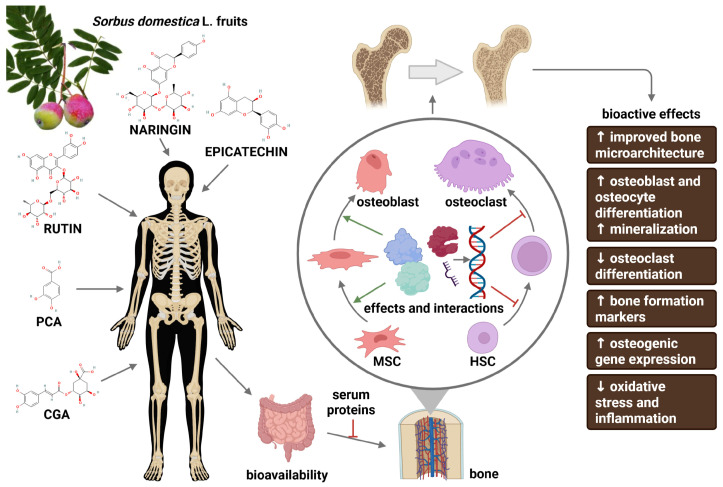
Summary of the bioactive effects of the most common polyphenols found in *Sorbus domestica* L. fruits on bone health. Bone cells regulate bone microstructure at multiple levels, from transcriptional and epigenetic regulation to post-translational modification of matrix proteins that control bone remodelling and mineralization (circled; details of the effects and interactions are given in Figure 2). Polyphenols can modulate these processes directly or indirectly. Cumulative evidence suggests that CGA, PCA, rutin, epicatechin, and naringin positively influence the bone microenvironment. In damaged bone microarchitecture, these compounds may attenuate bone loss and promote improved bone integrity through increased osteoblast and osteocyte differentiation and enhanced mineralization, while reducing oxidative stress, inflammation, and osteoclast differentiation. HSC—hematopoietic stem cells; MSC—mesenchymal stem cells. ↑—increased and ↓—decreased. Created with BioRender.com using chemical structures from PubChem (https://pubchem.ncbi.nlm.nih.gov); CIDs: 1794427, 72, 5280805, 442428, 72276.

Overall, Figure 1 summarizes the therapeutic effects of CGA, PCA, rutin, epicatechin, and naringin on bone biology.

## 4. Physicochemical Properties of Polyphenols and Interactions with Bone-Related and Signalling Mediators

For a phytochemical metabolite to exert a bioactive effect, it must reach the site of action to interact with target cells, either extracellularly or intracellularly. Polyphenols exhibit bioactivity through extracellular receptor interactions, triggering signalling pathways that enhance cellular function. For example, they may interact with G protein-coupled estrogen receptor 1 (GPER1) expressed in osteoblasts and osteocytes [34,119]. Cellular entry is shaped by serum proteins and membrane transporters that govern systemic distribution and intracellular availability [120]. Passive diffusion favours small, nonpolar molecules, while larger and more polar polyphenols show limited transcellular transport. Biotransformation and hydrolysis generate smaller, more nonpolar metabolites that often cross membranes more readily, potentially increasing cellular uptake relative to the parent compounds. In complex tissues such as bone, active transport through membrane carriers may be required [121]. Although direct uptake by osteoblasts, osteoclasts, or osteocytes has not been firmly established, studies suggest that transporters in the ABC and SLC (SLC21/SLC22) families could mediate polyphenol movement between intracellular compartments and the extracellular environment during mineralization [122]. 

**Figure 2:**
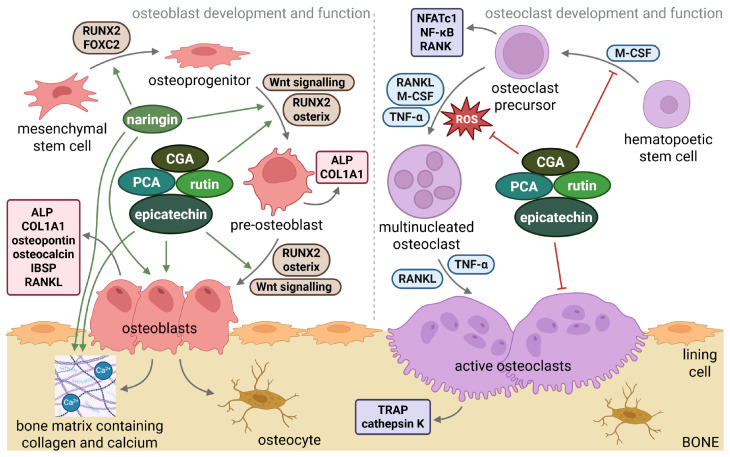
Interactions of the most common polyphenols found in *Sorbus domestica* L. fruits with the bone-related pathways and signaling mediators of osteoblast and osteoclast development. Osteoblast development begins with mesenchymal stem cells that differentiate into osteoprogenitor cells, pre-osteoblasts and active osteoblasts. Osteoblasts produce mineralized organic matrix to form new bone, which is accompanied by the production of marker molecules such as ALP, osteocalcin. The process is influenced by signalling pathways (such as Wnt) and gene regulation (such as Runx2). Osteoclast development originates from hematopoietic stem cells, involving specific differentiation of precursors and their fusion into multinucleated cells. The process is regulated by signalling molecules such as M-CSF, RANKL, and is encouraged by oxidative stress and inflammation. Active osteoclasts resorb bone and express functional markers such as TRAP, cathepsin K. The polyphenols described in this article interact with both signalling pathways and mediators of osteoblast and osteoclast development. Blunt red arrows indicate an inhibitory effect, sharp green arrows designate a stimulatory effect. Grey arrows indicate the connection of individual mechanisms. ALP—alkaline phosphatase; CGA—chlorogenic acid; COL1A1—collagen type I alpha 1; FOXC2—forkhead box C2; IBSP—integrin-binding sialoprotein; M-CSF—macrophage colony-stimulating factor; NF-κB—nuclear factor kappa B; NFATc1—nuclear factor of activated T-cells; PCA—protocatechuic acid; RANK—receptor activator of nuclear factor kappa-Β; RANKL—receptor activator of nuclear factor kappa-Β ligand; ROS—reactive oxygen species; RUNX2—runt-related transcription factor 2; TNF-α—tumor necrosis factor-alpha; TRAP—tartrate-resistant acid phosphatase. Created with BioRender.com.

5-CQA (molecular weight; MW = 354.31 g/mol) is relatively large for efficient passive membrane diffusion, and its polarity at physiological pH further reduces transcellular permeability [123,124]. It undergoes deprotonation with pKa_1_ ≈ 3.5 (carboxyl group) and a second deprotonation around pKa_2_ ≈ 8.4–11 [125]. Consequently, transcellular transport of 5-CQA and its metabolites may be limited by size and polarity. GCA has been found to affect important signalling pathways and processes in bone, specifically increasing the expression of osteogenesis genes for frizzled-related protein and pyruvate dehydrogenase kinase 4 and inhibiting the expression of osteoclastogenesis genes such as the genes for asporin and cytokine-like 1. CGA also reduced canonical Wnt/β-catenin signalling but increased noncanonical Wnt/Ca^2+^ signalling (Figure 2) [126]. In silico molecular docking indicates drug-like properties of 5-CQA and its potential intracellular actions via interactions with TNF-α, RUNX2, and osteocalcin, but these predictions require experimental validation [127]. 

PCA (MW = 154.12 g/mol) is mainly monoanionic at physiological pH, with carboxyl deprotonated (pKa_1_ ≈ 4–4.5) and phenolic OH groups largely protonated (pKa_2_ ≈ 8.5–12), resulting in high polarity and limited passive diffusion at pH 7.4 [72,128,129]. A recent in vitro analysis using human MSCs revealed a positive effect of PCA on osteoblast differentiation, as demonstrated by increased expression of *SPP1*, *RUNX2* and *ALPL* genes and elevated Ca deposition [130]. Molecular docking showed that PCA binds IGF-1, potentially activating AKT serine/threonine kinase 1 (AKT1) signalling in osteoprogenitor cells and promoting osteoblast proliferation and differentiation [76]. Protocatechuic aldehyde, a related less-polar compound, can activate signalling via G protein-coupled estrogen receptor (GPER) and upregulate GPER expression, supporting its pharmacological effects [131]. In addition, according to BindingDB (www.bindingdb.org), PCA can inhibit carbonic anhydrase 2 enzyme, which is essential for bone resorption and osteoclast differentiation [132]. 

Rutin (MW = 610.52 g/mol [88]) is a relatively large polyphenolic glycoside. Its hydrophilic character limits passive diffusion across membranes, and its ionization depends on the presence of multiple phenolic OH groups and the glycosidic bonds. Therefore, there is no universal pKa value [133]. At pH 7.4, rutin is largely polar and weakly ionized, depending on deprotonation. Rutin increased the expression of *Runx2* gene and the activity of ALP in MC3T3-E1 cells [134], and positive regulated the Wnt signalling pathway in MSCs (Figure 2) [93]. It also inhibited osteoclast formation by decreasing ROS and TNF-α by inhibiting NF-κB activation [85]. In addition, molecular docking suggests possible binding affinity of rutin with TRAF-6 and BCL2, regulators of inflammation and apoptosis [135]. 

Epicatechin (MW = 290.27 g/mol) is moderately polar due to its multiple hydroxyl groups [136]. At pH 7.4, it is not strongly ionized, and its polarity and hydrogen-bonding capacity may limit passive diffusion, making transporter-mediated uptake plausible. A study of human epithelial cells demonstrated unsaturated uptake, indicating that diffusion (or paracellular routes) may dominate under these conditions, although diffusion and carrier-mediated transport could coexist in other contexts [137]. Epicatechin demonstrated positive effect on the osteogenic markers such as RUNX2, BMP2, and ALP. It also promoted osteogenesis by blocking NFATc-1, NF-κB and inhibited the RANKL-RANK interaction [107,138]. Molecular docking suggests high affinity for GPER, implying potential GPER-mediated signalling in bone cells, but experimental validation is required [139]. According to BindingDB, epicatechin can inhibit fatty acid synthase and aldose reductase enzymes, which in turn enhances bone formation and affects secondary diabetic complications (including diabetic bone disease) [8,140].

Naringin (MW = 580.54 g/mol) is a polar polyphenol with multiple hydroxyl groups [141]. Its pKa values (~7.05–8.84) indicate that it can exist in both ionized and non-ionized forms at pH 7.4. Membrane passage may occur via active transport, although passive diffusion is possible in human colorectal adenocarcinoma cells (Caco-2 cells) [142]. Naringin promoted osteogenic differentiation of BMSCs by upregulating Forkhead box C2 gene (Foxc2) expression via the hedgehog signalling pathway [143]. Foxc2 is known to stimulate osteoblast differentiation of mesenchymal cells and preosteoblasts by activating the canonical Wnt/β-catenin signalling (Figure 2). In silico analyses showed strong binding to bone-regulatory proteins, including IL-6, TNF-α, BCL2, AKT1, estrogen receptor 1 (ESR1), and signal transducer and activator of transcription 3 (STAT3) [144]. 

Possible polyphenol mechanisms in bone are being actively studied. In vitro, compounds such as CGA can bind directly to free Ca^2+^ or hydroxyapatite, possibly coordinating Ca^2+^ in hydroxyapatite to facilitate association with the mineral phase [35,145]. At physiological pH, deprotonation creates charged oxygen sites that can chelate metals, often via catechol groups. Chelation of Ca-polyphenols may occur in osteoblasts, facilitating incorporation into bone. An alternative mechanism for the distribution of polyphenols into hydroxyapatite involves their binding to phospholipids to form matrix vesicles that deliver Ca^2+^ to hydroxyapatite and polyphenols to the mineralizing matrix [122,146].

Polyphenols can also interact with COL1A1, with cross-linking enhancing mechanical properties of the bone matrix. Covalent bonds may dominate under acidic conditions, while noncovalent interactions predominate at alkaline pH [36,147]. Most of the data are obtained in vitro, and in vivo cross-linking remains poorly studied. It is possible that polyphenols interact with bone matrix directly from the circulation and they are redeposited into bone. During remodelling, osteoclast activity creates a mildly acidic microenvironment (pH~6.8), which can promote deconjugation and alter polyphenol binding near the mineralization front [148]. 

In general, bioactive effects of polyphenols are modulated by pH, temperature, pressure, environmental factors, and the cellular microenvironment. An important factor to consider in experiments is the use of fetal bovine serum (FBS) in cell culture medium. FBS components can interact with polyphenols. Tang et al. [149] showed that hydroxylated resveratrol analogues are destabilized by FBS, while glycosylation and methoxylation enhance stability. These interactions in the serum may mask antioxidant effects and reduce polyphenol bioavailability in vitro. Polyphenols bind strongly to proteins, causing conformational and physicochemical changes [150]. Albumin, a major plasma protein, can interact with rutin to enable electrostatic interactions and stabilise it in circulation [151]. Similar albumin–polyphenol interactions have been documented for CGA, PCA, epicatechin, and naringin [152,153,154,155]. Protein-polyphenol interactions are context-dependent. They can reduce polyphenol bioavailability when dietary proteins bind to them and limit absorption, but they can also stabilize or form complexes that alter a protein’s hydrophobic-hydrophilic balance and solubility, potentially enhancing protein structure [150,156,157].

Dietary components can modulate polyphenol bioaccessibility and bioactivity, as is the case for *Sorbus domestica* L. fruits. For example, the matrix extract of black chokeberry contained more polyphenols than the isolated extract (2.3×), but the isolated polyphenols showed greater bioactivity. Digestion significantly reduced the polyphenol content and bioactivity in the matrix extract [158]. These findings highlight the need to study polyphenols from *Sorbus domestica* L. fruits in relevant contexts, including digestion, serum interactions, and protein binding to understand their potential osteoprotective mechanisms.

## 5. Polyphenol-Polyphenol Interactions

Understanding the interactions among polyphenols is essential for maximizing their health benefits. The effects of polyphenol combinations are dose- and concentration-dependent. Within certain concentration ranges, specific polyphenol pairs can exhibit synergistic interactions that enhance bioactivity, including antioxidant capacity and anti-inflammatory responses. By contrast, at higher concentrations, polyphenols may produce antagonistic or inhibitory effects that diminish overall efficacy and potentially alter bioavailability and metabolic outcomes [159,160]. Given the limited evidence on the modulatory effects of polyphenol-polyphenol interactions on bone health, this section focuses on investigating combinations of polyphenols from *Sorbus domestica* L. fruits (CGA, PCA, rutin, epicatechin, naringin, quercetin) and their bioactivities (Table 6).

Antioxidant activity is a critical parameter in bone health, as maintaining redox homeostasis supports osteoblast and osteoclast function and helps prevent oxidative stress-induced bone fragility [161]. In addition, the potency of polyphenols, often assessed using assays such as Ferric Reducing Antioxidant Power (FRAP) and 2,2-diphenyl-1-picrylhydrazyl (DPPH), and higher in vitro antioxidant activity may reflect a greater ability to mitigate oxidative damage to bone tissue. Notably, several polyphenol-polyphenol interactions have been shown to exhibit synergistic or additive enhancement of antioxidant activity. For example, CGA (0.1 mM) in combination with PCA (0.1 mM) resulted in a synergistic increase in radical scavenging activity as measured by DPPH, while CGA (600 μM) with rutin (150 μM) resulted in an additive interaction [162,163]. Additional additive antioxidant effects have been reported for CGA with rutin and epicatechin with rutin based on both DPPH and FRAP [164,165]. Although these in vitro antioxidant interactions do not directly demonstrate bone health outcomes, the increased redox-modulating potential they represent may provide a plausible mechanistic basis for indirect benefits to bone health—particularly in contexts where oxidative stress causes bone loss. Consequently, the synergistic effects of polyphenol combinations in antioxidant activity could help maintain the balance of bone remodelling under oxidative stress. However, further studies focusing directly on bone are needed to confirm these indirect conclusions.

The beneficial effects of the aforementioned polyphenols also include anticancer activity, particularly in various cancer cell lines. This dual property may be critically linked to bone health, especially in the context of cancer, where the pathological state of malignant cells alters the normal physiological processes of bone remodelling [166]. Cancer cells often reside in a microenvironment with elevated ROS. While moderate ROS may support cancer cell survival and metabolic adaptation, higher levels of ROS may trigger cellular stress responses that promote autophagy and, in some contexts, apoptosis, reflecting the dual role of ROS and autophagy in cancer progression [167]. For example, studies have shown that the combination of naringin, rutin, and quercetin not only synergistically promoted ROS production but also triggered apoptosis in HeLa cervical cancer cells, suggesting that polyphenols can exploit the vulnerability of cancer cells while potentially protecting normal cells from similar oxidative stress [168]. Furthermore, interactions between epicatechin and naringenin in colorectal cancer cells have demonstrated cytotoxicity while exhibiting less deleterious effects in normal colon cell lines, highlighting the selective nature of polyphenols in targeting diseased tissues [169]. This selective induction of apoptosis in cancer cells could have a significant impact on bone health, particularly in patients with cancer-induced bone loss or metastasis [167]. Oxidative stress produced by cancer cells can disrupt bone remodelling, contributing to osteolytic lesions and other skeletal complications [166]. By promoting apoptosis of cancer cells, polyphenols may help alleviate cancer-related bone deterioration.

**Table 6:** The combination of CGA, PCA, rutin, epicatechin, naringin, and quercetin and their dose-dependent interaction type.

Combination	Concentration	Obtained Results	References
CGA PCA	0.1 mM 0.1 mM	SYNERGISTIC -enhanced radical scavenging capacity	[162]
CGA Rutin	600 μM 150 μM	ADDITIVE -antioxidant activity	[163]
CGA Rutin	100 μg/mL 100 μg/mL	SYNERGISTIC -inhibition of thyroid peroxidase activity	[164]
CGA Rutin	Not specified	ADDITIVE -enhanced antioxidant activity	[165]
Epicatechin Rutin	Not specified	ADDITIVE -enhanced antioxidant activity	[165]
Naringin Rutin Quercetin	170 μM 8 μM 8 μM	SYNERGISTIC -anticancer effects, enhanced ROS, apoptosis of HeLa cervical cancer cells	[168]
Epicatechin Naringin	20 µM 25 µM	SYNERGISTIC -increased cell death, anoikis in HCT-116 and T84 cancer cell lines	[169]
CGA Quercetin	600 μM 150 μM	SYNERGISTIC -antioxidant activity	[163]

Abbreviations: CGA—chlorogenic acid; PCA—protocatechuic acid; ROS—reactive oxygen species.

## 6. Polyphenols and Epigenetic Modifications of Bone-Related Targets

Epigenetic regulation, including DNA methylation, histone modifications, and non-coding RNAs (miRNA, lncRNA) plays a critical role in balancing osteoblast and osteoclast activity and bone health. Understanding these epigenetic modifications may enable the diagnosis and therapy of osteoporosis and osteoarthritis [170,171]. The epigenetic methylation of CpG dinucleotides plays an important role in the regulation of *ALPL* expression in cells of the osteoblastic lineage and specifically in gene silencing during the transition from osteoblasts to osteocytes [172]. Posttranslational modifications of histones also shape gene expression. Histone acetylation generally promotes transcription (e.g., higher osteocalcin expression with increased acetylation), whereas deacetylation can suppress RUNX2-target gene transcription and osteoblast differentiation. MicroRNAs affect the bone metabolism by regulating the differentiation, proliferation, apoptosis, and autophagy of osteoblasts, osteoclasts, and osteocytes. Some miRNAs, such as miRNA-33-5p and miRNA-433-3p, promote osteoblastic differentiation via the downregulation of osteoblastogenesis inhibitors or through RUNX2 activation (miRNA-194). In contrast, other miRNAs, such as miRNA-125b, miRNA-375, and miRNA-133a-5p, can inhibit osteoblastogenic differentiation by acting directly or indirectly on RUNX2. Additionally, there are several miRNAs, such as miRNA-34a or miRNA-124-3p, miRNA-125a-5p that inhibit osteoclastogenesis by blocking transforming growth factor-beta-induced factor 2 or suppressing NFATc1 expression, respectively. Conversely, other miRNAs can promote osteoclastogenesis by acting on relevant signalling pathways (e.g., miRNA-21-5p, miRNA-29, miRNA-183-5p) [173]. Several miRNAs have been studied in relation to the regulation of osteogenesis in osteoporosis, such as let-7a-5p, miR-10b, miR-221-5p, miR-203-3p, miR-590-5p, miR-1297, miR-9-5p, miR-129-5p, miR-135a-5p, miR-338-3p, and miR-205 [174]. Furthermore, miR-21, miR-133a, and miR-146a were found to be related to BMD or fragility fractures in postmenopausal women [7]. 

Polyphenols may influence bone health by reprogramming epigenetic pathways, offering potential strategies against osteoporosis. However, the evidence in bone biology is limited and much information comes from non-bone contexts. CGA can reduce DNA methylation through DNA methyltransferase inhibition in leukemic cells [175]. Anthocyanins, whose major metabolite is PCA, have been linked to multiple epigenetic mechanisms in bone cells via non-coding RNAs (including lncRNA), with anthocyanin-rich foods showing epigenetic effects in osteoblasts and osteocytes, such as changes in histone acetylation/deacetylation [176,177]. Epicatechin has been shown to decrease the activity of CBP/p300 histone acetyltransferases and histone deacetylase (HDAC4), enzymes that are upregulated in inflammatory human monocytes induced by high glucose [178]. Naringin/naringenin exerts anticancer, antioxidant, and anti-inflammatory effects through diverse epigenetic mechanisms, potentially via regulation of non-coding RNAs (miRNA, lncRNA, circRNA) [179]. Finally, 3,4-dihydroxytoluene, a rutin metabolite, inhibits the bromodomain-recognition activity of p300, a histone acetyltransferase, thereby contributing to the suppression of non-alcoholic fatty liver disease progression [180]. 

Notably, naringin has been investigated for its potential to promote osteoblastogenesis, in part via modulation of miRNA pathways. In BMSCs cultured in osteogenic medium, 100–1000 μM of naringin for 24 h reduced expression of Ski (a negative regulator of osteoblast differentiation) and markedly upregulated miR-26a, likely by targeting the Ski mRNA 3′ UTR [37]. In another study, naringin (1 μM for 48 h) also reduced the protein levels of PPARγ in BMSCs and increased miR-20a expression. Transfection of miR-20a further reduced PPARγ protein levels, and co-treatment of miR-20a with naringin partially blocked this effect, suggesting that naringin acts through miR-20a–mediated regulation of PPARγ [181]. Since *Sorbus domestica* L. fruits contain all these polyphenols, they may affect bone health through epigenetic mechanisms as well, but conclusive bone-specific evidence is still lacking.

## 7. Conclusions

A growing body of research on polyphenols underscores their potential role in maintaining and improving bone health. The diverse mechanisms through which these metabolites function—ranging from antioxidant and anti-inflammatory activities to modulation of osteoblast and osteoclast differentiation—suggest that they could be a valuable addition to nutritional strategies aimed at preventing bone loss. Preliminary evidence supporting the osteoprotective properties of CGA, PCA, rutin, epicatechin, and naringin, found in *Sorbus domestica* L. fruits, highlights the need for further investigation of their bioavailability and metabolic pathways to fully understand their implications for human health. Current studies have largely focused on preclinical models; therefore, well-designed clinical trials are essential to verify the efficacy of these polyphenols. As metabolic bone diseases, including osteoporosis and diabetes mellitus, represent a significant public health challenge, especially in the aging population, the integration of *Sorbus domestica* L. fruits into dietary recommendations could represent a practical approach to improve bone health and thereby reduce fracture risk. Considering the maturation of *Sorbus domestica* L. fruits, the trade-off between polyphenol content and sensory properties suggests that the optimal period from harvest to consumption is approximately two weeks after harvest, with the exact time being influenced by the stage of ripening, storage duration and desired sensory properties. Studies also suggest that unripe fruit may have higher polyphenol content and stronger antioxidant activity. Therefore, it is processed into fermented products such as vinegar. Future research should also explore the synergistic effects of these polyphenols in combination with other dietary components, as well as their potential epigenetic influences on bone metabolism, to further elucidate their role in promoting bone health.

## Figures and Tables

**Table 1:** Studies utilizing CGA and monitoring its effects on bone health.

Research Type Model	Research Model	Applied Treatment	Obtained Results	References
In vitro	RAW264.7 macrophage cell line	CGA: 0.1–10 μg/mL for 7 days applied with RANKL	↓ TRAP activity	[29]
In vitro	MG-63 murine monocyte cells	CGA: 0.1–10 μg/mL 2 days to assess viability, and 21 days to assess mineralization	↑ proliferation, ALP activity; ↑ mineralization	[29]
In vitro	BMSC	CGA: 0.1–10 μM for 3 and 7 days	↑ proliferation; ↑ differentiation (ALP activity)	[55]
In vitro	MC3T3-E1 preosteoblasts	CGA: 50 μM for 60 min prior to TNFα stimulation	↑ *Il-6* mRNA levels	[58]
In vitro	MC3T3-E1 preosteoblasts	CGA: 50, 100 μM for 3 h in DEX-induced conditions	↑ viability; ↓ caspase activity; ↓ ROS, mitochondrial apoptosis	[21]
In vitro	MLO-Y4 osteocytes like cell line	CGA: 10, 100 μM with prior DEX-treatment	↑ gene expression of *Runx2*, *Bglap*, β-catenin, and *Cx43*; ↓ ROS, apoptosis rate	[56]
In vitro	Mouse BMMs	CGA: 50 μg/mL with RANKL and M-SCF for 4 day (TRAP-staining) and 2 days (gene expression analysis)	↓ TRAP activity; ↓ *Nfatc1* and *Trap* mRNA levels	[59]
In vivo	OVX rats (n = 24)	CGA: 50, 75 mg/day for 8 weeks	↑ tibial Tb.N, Tb.Th and BV/TV	[29]
In vivo	OVX rats (n = 60)	CGA: 27, 45 mg/kg/day for 12 weeks	↑ total femur BMD, Tb.N, Tb.Th, BV/TV, Conn.D; ↑ALP, osteocalcin	[55]
In vivo	OVX rats (n = 18)	CGA: 45 mg/kg/day for 12 weeks	↑ total femur BMD, Tb.N, Tb.Th, BV/TV, SMI	[64]
In vivo	OVX rats (n = 81–90)	CGA: at 100 mg/kg/day for 4 weeks	↑ femur trabeculae width	[63]
In vivo	Femoral head necrosis model rats (n = 60)	CGA: 20 mg/kg/day for 4 weeks	↑ femoral head BMD	[57]
In vivo	DEX-induced osteoporotic mice (n = 40)	CGA: 0.5 mg/kg/day for 4 weeks	↑ femoral Tb.Th, BMD; ↓ femoral Tb.Sp; ↑ ALP, P1NP	[56]
In vivo	LPS-induced bone erosion mice (n = not specified)	CGA from *Gardenia jasminoides*: 10 mg/kg/day for 8 days	↑ femoral BV/TV, Tb.Th	[59]
Clinical trial	Participants with BMI > 25 kg/m^2^ (n = 33)	green coffee bean extract: 400 mg twice a day for 8 weeks	↑ INS sensitivity; ↓ FBG; ↓ body weight	[67]
Clinical trial	Participants with BMI >25 kg/m^2^ (n = 105)	GCB70® (70% 5-CQA, 30% 3-CQA): 500 mg twice a day for 12 weeks	↑ HDL; ↓ FBG, HbA1c; ↓ TSH, LDL	[68]
Clinical trial	Healthy participants (n = 42)	Polyphenol-rich lettuce 100 g/day for 12 days	↑ vitamin D; ↓ LDL, PTH	[69]

Abbreviations: ↑—increased; ↓—reduced; ALP—alkaline phosphatase; BMD—bone mineral density; BMI—body mass index; BMM—bone marrow-derived macrophages; BMSC—bone marrow–derived mesenchymal stem cells; *Bglap*—gene for bone gamma-carboxyglutamate protein.; BV/TV—bone volume fraction; Conn.D—connectivity density; CGA—chlorogenic acid; DEX—dexamethasone; FBG—reduced fasting blood glucose; HbA1c—glycosylated haemoglobin; HDL—high-density lipoprotein; *Il-6*—interleukin-6 gene; INS—insulin; LDL—low-density lipoprotein; *Nfatc1*—nuclear factor of activated T-cells gene, cytoplasmic 1; OVX—ovariectomized; PTH—parathyroid hormone; P1NP—procollagen type 1 *N*-terminal propeptide; RANKL—receptor activator of nuclear factor kappa-Β ligand; ROS—reactive oxygen species; *Runx2*—runt-related transcription factor 2 gene; SMI—structure model index; Tb.N—trabecular number; Tb.Sp—trabecular separation; Tb.Th—trabecular thickness; TRAP—tartrate-resistant acid phosphatase; *Trap*—tartrate-resistant acid phosphatase gene; TSH—thyroid-stimulating hormone.

**Table 2:** Studies using PCA to assess its effects on bone health.

Research Type Model	Research Model	Applied Treatment	Obtained Results	References
In vitro	Primary human osteoblasts	PCA: 50, 100 μM for 12 days	↑ *SPP1*, *IBSP*, *ALPL*, *BMP6*; ↑ IL-1β	[75]
In vitro	MC3T3-E1 (preosteoblast cell line) treated with DEX	PCA: 12.5, 25, and 50 μmol/L for 24 h and 7 days	↑ proliferation; ↑ ALP activity; ↑ SOD, GSH; ↓ ROS, MDA	[76]
In vitro	RAW264.7 macrophage cell line	PCA: 8 μM for 2 h prior treated with RANKL	↑ SOD, GPx, GSH; ↓ ROS; ↓ TRAP activity; ↓ *Trap*, *Traf6*, *c-Src*, *Mmp9*, cathepsin mRNA levels	[77]
In vitro	RAW264.7 macrophage cell line	PCA: 8 μM for 2 h prior treated with RANKL	↓ TRAP activity; ↓ NFATc1, NF-κB protein levels	[78]
In vitro	Murine C3H10T1/2 MSC cells	PCA: 80 μM for 6- or 9-day in osteogenic induction conditions	↑ ALP activity; ↑ mineralization; ↑ *Runx2*, *Alpl*, *Sp7* mRNA levels	[79]
In vivo	OVX mice (n = 30)	PCA: 20 mg/kg/day for 12 weeks	↑ tibial trabecular BMD, Tb.N, and BV/TV; ↓ tibial Tb.Sp; ↑ OPG; ↓ RANKL; ↓ *Nfatc1*, *Traf6* mRNA levels	[31]
In vivo	Alcohol-induced osteopenic mice (n = 20)	PCA: 50 mg/kg for 40 days	↑ tibial trabecular BMD, BV/TV, Tb.N	[80]
Clinical trial	Participants with T2DM (n = 58)	Anthocyanin (major metabolite: PCA), 160 g twice a day for 24 weeks	↑ INS sensitivity; ↓ FBG, TAG; ↓ TNF-α, IL-6	[82]

Abbreviations: ↑—increased; ↓—reduced; ALP—alkaline phosphatase; *ALPL*—alkaline phosphatase gene; BMD—bone mineral density; *Bmp6*—bone morphogenetic protein 6 gene; BV/TV—bone volume fraction; *c-Src*—proto-oncogene tyrosine-protein kinase Src gene; DEX—dexamethasone; FBG—reduced fasting blood glucose; GPx—glutathione peroxidases; GSH—glutathione; *Ibsp*—integrin-binding sialoprotein gene; IL-1β—interleukin-1 beta; IL-6—interleukin-6; INS—insulin; MDA—malondialdehyde; *Mmp9*—matrix metalloproteinase-9 gene; NFATc1—nuclear factor of activated T-cell; *Nfatc1*—nuclear factor of activated T-cells gene; NF-κB—nuclear factor kappa B; OPG—osteoprotegerin; OVX—ovariectomized; PCA—protocatechuic acid; RANKL—receptor activator of nuclear factor kappa-Β ligand; ROS—reactive oxygen species; *Runx2*—runt-related transcription factor 2 gene; SOD—superoxide dismutase; *Sp7*—Sp7 transcription factor 7 gene (osterix); *Spp1*—osteopontin gene; TAG—triacylglycerol; Tb.N—trabecular number; Tb.Sp—trabecular separation; Tb.Th—trabecular thickness; TNF-α—tumor necrosis factor-alpha; *Traf6*—TNF receptor-associated factor-6 gene; TRAP—tartrate-resistant acid phosphatase; *Trap*—tartrate-resistant acid phosphatase gene; T2DM—type 2 diabetes mellitus.

**Table 3:** Studies utilizing rutin to assess its effects on bone health.

Research Type Model	Research Model	Applied Treatment	Obtained Results	References
In vitro	PDLSCs	Rutin: 1 × 10^−6^ mol/L for 3 days proliferation assay, and 7 days for differentiation	↑ proliferation; ↑ osteogenic differentiation, ALP activity; ↑ COL1A1, ALP, RUNX2 and SPP1 mRNA and protein levels	[91]
In vitro	MG-63 human osteosarcoma cell lines	Rutin: 10 and 25 μg/mL in 3, 5, and 7 days for proliferation, collagen synthesis and mineralization, respectively	↑ proliferation, ALP activity; ↑ collagen production; ↑ mineralization	[92]
In vitro	MSCs	Rutin: 50 mg/mL for 24 h	↑ COL1A1, RUNX2, osteocalcin, osteopontin mRNA and protein levels; ↑ mineralization	[93]
In vitro	Mouse bone marrow cells	Rutin: 20 μM for 3 days with M-CSF and RANKL	↓ TRAP activity; ↓ ROS; ↓ TNF-α	[94]
In vivo	OVX mice (n = 30)	Rutin: 50 mg/kg for 4 or 8 weeks	↑ femoral BV/TV, Tb.Th; ↓ femoral Tb.Sp.; ↓ ALP, CTx; ↓ IL-1β, IL-6, and TNF-α	[32]
In vivo	OVX rats (n = 40)	Rutin: 10 mg/kg/day for 3 months	↑ total femur BMD; ↓ osteocalcin, vitamin D; ↓ IL-6, TNF-α, IFN-γ	[95]
In vivo	Bone defect rabbit model (tooth removal) (n = 6)	Rutin: 2.5 mg mixed with 900 mg of demineralized freeze-dried bone allograft left in for 6 weeks	↑ *COL3A1* mRNA levels; ↓ MMPs mRNA levels	[96]
Clinical trial	Participants with T2DM (n = 50)	Rutin: 500 mg/day for 3 months	↑ INS sensitivity; ↑ HDL; ↓ FBG, HbA1c, INS; ↓ LDL; ↓ IL-6 and MDA	[97]
Clinical trial	Participants with T2DM (n = 50)	Rutin: 500 mg/day for 3 months	↑ SOD, GPx	[98]

Abbreviations: ↑—increased; ↓—reduced; ALP—alkaline phosphatase; BMD—bone mineral density; BV/TV—bone volume fraction; COL1A1—collagen type I alpha1; *COL3A1*—collagen type III alpha 1 gene; CTx—*C*-terminal telopeptide of type 1 collagen; FBG—reduced fasting blood glucose; GPx—glutathione peroxidases; HbA1c—glycosylated haemoglobin; HDL—high-density lipoprotein; IFN-γ—interferon-gamma; IL-1β—interleukin-1 beta; IL-6—interleukin-6; INS—insulin; LDL—low-density lipoprotein; M-CSF—macrophage colony-stimulating factor; MDA—malondialdehyde; MMPs—matrix metalloproteinase genes; MSC—mesenchymal stem cells; OVX—ovariectomized; PDLSCs—human periodontal ligament stem cells; RANKL—receptor activator of nuclear factor kappa-Β ligand; ROS—reactive oxygen species; RUNX2—runt-related transcription factor 2; SOD—superoxide dismutase; Tb.Sp—trabecular separation; Tb.Th—trabecular thickness; TNF-α—tumor necrosis factor-alpha; TRAP—tartrate-resistant acid phosphatase; T2DM—type 2 diabetes mellitus.

**Table 4:** Studies utilizing epicatechin to assess its effects on bone health.

Research Type Model	Research Model	Applied Treatment	Obtained Results	References
In vitro	RAW264.7 macrophage cell line	ECAP: 50 and 100 μg/mL for 24 h and 5 days, pre-treated with RANKL	↓ TRAP activity; ↓ MMP9, NFATc1 mRNA and protein levels	[30]
In vitro	MSC-hBM	Epicatechin: 1 and 100 µM for 72 h	↑ *RUNX2*, *SPARC* mRNA levels	[107]
In vitro	Rat osteoblasts	Epicatechin: 25 and 50 μg/mL, for 48 h, 7 days, and 20 days	↑ differentiation, ALP activity; ↑ hydroxyapatite formation; ↑ mineralization	[108]
In vivo	OVX mice (n = 32)	ECAP: 100 mg/kg/day for 4 weeks	↑ femoral Tb.N, Tb.Th, BV/TV; ↓ femoral Tb.Sp; ↓ osteocalcin, CTx	[30]
Clinical trial	Healthy women (≥65 y) and men (≥60 y) (n = 21,442)	Daily cocoa extra supplement contained a total of 500 mg/day of flavanols including 80 mg/day of epicatechin for 3.6 years (median)	No effect on a risk of incident clinical fracture	[109]
Clinical trial	Healthy participants (n = 35)	Epicatechin: 100 mg/day for 4 weeks	↑ INS sensitivity; ↓ INS	[110]
Clinical trial	Participants with (BMI) > 18.5 and <30 kg/m^2^ (n = 20)	Epicatechin: 1 mg/kg once and after 4 h blood chemistry tests	↓ plasma glucose, TAG	[111]
Clinical trial	Postmenopausal women with T2DM (n = 93)	Flavonoid-enriched chocolate: epicatechin 90 mg combined with flavan-3-ols and isoflavones	↑ INS sensitivity; ↑ HDL; ↓ INS	[112]

Abbreviations: ↑—increased; ↓—reduced; ALP—alkaline phosphatase; BMI—body mass index; BV/TV—bone volume fraction; CTx—*C*-terminal telopeptide of type 1 collagen; ECAP—epicatechin 3-O-β-D-allopyranoside; HDL—high-density lipoprotein; INS—insulin; MMP9—matrix metalloproteinase-9; MSC-hBM—human mesenchymal stem cells; NFATc1—nuclear factor of activated T-cells; OVX—ovariectomized; RANKL—receptor activator of nuclear factor kappa-Β ligand; *RUNX2*—runt-related transcription factor 2 gene; *SPARC*—secreted protein acidic and cysteine rich gene; TAG—triacylglycerol; Tb.N—trabecular number; Tb.Sp—trabecular separation; Tb.Th—trabecular thickness; TRAP—tartrate-resistant acid phosphatase; T2DM—type 2 diabetes mellitus.

**Table 5:** Studies utilizing naringin and monitoring its effects on bone health.

Research Type Model	Research Model	Applied Treatment	Obtained Results	References
In vitro	MC3T3-E1 preosteoblasts	Naringin: 0.5–1 μM for 24 h, 14 days, and 21 days	↑ proliferation; ↑ differentiation, ALP activity; ↑ mineralization; ↑ RUNX2, COL1A1, osteopontin mRNA and protein levels	[117]
In vivo	OVX mice (n = 40)	Naringin: 30 mg/kg/day for 12 weeks	↑ femoral BMD, BV/TV, Tb.N, Tb.Th; ↓ femoral Tb.Sp; ↑ P1NP, osteocalcin; ↓ CTx	[117]
Clinical trial	Participant with diabetes (n = 1)	Naringin: 150 mg three times daily for 8 weeks	↑ INS sensitivity; ↓ INS	[118]

Abbreviations: ↑—increased; ↓—reduced; ALP—alkaline phosphatase; BMD—bone mineral density; BV/TV—bone volume fraction; COL1A1—collagen type I alpha 1; CTx—*C*-terminal telopeptide of type 1 collagen; INS—insulin; OVX—ovariectomized; P1NP—procollagen type 1 *N*-terminal propeptide; RUNX2—runt-related transcription factor 2; Tb.N—trabecular number; Tb.Sp—trabecular separation; Tb.Th—trabecular thickness.

## Data Availability

No new data were created or analyzed in this study.

## Abbreviations

3-HPAA	3-(3-hydroxyphenyl) acetic acid
AKT1	AKT serine/threonine kinase 1
Al	aluminum
ALP	alkaline phosphatase
*ALPL*	alkaline phosphatase gene
AP-1	activator protein 1
B	boron
BAX	BCL2 associated X
Bche	butyrylcholinesterase
BCL2	B-cell lymphoma 2
*Bglap*	bone gamma-carboxyglutamate protein gene
BMD	bone mineral density
BMI	body mass index
BMMs	bone mouse marrow-derived macrophages
*BMP6*	bone morphogenetic protein 6 gene
BMSC	bone mesenchymal stem cells
BV/TV	bone volume fraction
Ca	calcium
CAT	catalase
CGA	chlorogenic acid
COL1A1	collagen type I alpha 1
*COL1A1*	gene encoding collagen type I alpha 1
*COL3A1*	collagen type III alpha 1 gene
Conn.D	connectivity density
*CPT1β*	carnitine palmitoyltransferase 1 beta gene
*c-Src*	proto-oncogene tyrosine-protein kinase Src gene
CTx	*C*-terminal telopeptide of type 1 collagen
Cu	copper
DEX	dexamethasone
DPPH	2,2-diphenyl-1-picrylhydrazyl
dw	dry weight
ECAP	epicatechin 3-O-β-D-allopyranoside
ESR1	estrogen receptor 1
FBG	fasting blood glucose
FBS	fetal bovine serum
Fe	iron
FRAP	Ferric Reducing Antioxidant Power
fw	fresh weight
*GLUT4*	glucose transporter type 4 gene
GPER	G protein-coupled estrogen receptor
GPER1	G protein-coupled estrogen receptor 1
GPx	glutathione peroxidases
GSH	glutathione
HbA1c	glycosylated haemoglobin
HDL	high-density lipoprotein
HO-1	hemoxygenase 1
HOMA-IR	Homeostasis Model Assessment of Insulin Resistance
HPPA	3-(3-hydroxyphenyl) propionic acid
*IBSP*	integrin-binding sialoprotein gene
IBSP	integrin-binding sialoprotein
IFN-γ	interferon-gamma
IGF-1	insulin-like growth factor 1
IL-1β	interleukin-1 beta
IL-6	interleukin-6
INS	insulin
K	potassium
Keap1	Kelch-like ECH-associated protein 1
LDL	low-density lipoprotein
LPS	lipopolysaccharide
M-CSF	macrophage colony-stimulating factor
MDA	malondialdehyde
*Mmp9*	matrix metalloproteinase-9 gene
MMP9	matrix metalloproteinase-9
*MMPs*	matrix metalloproteinase genes
MPO	myeloperoxidase
MSC	mesenchymal stem cells
MSC-hBM	human mesenchymal stem cells
*Nfatc1*	nuclear factor of activated T-cells gene
NFATC1	nuclear factor of activated T-cells
NF-Κb	nuclear factor kappa B
Nrf2	erythroid 2-related factor transcription factor
OPG	osteoprotegerin
OVX	ovariectomized
P	phosphorus
P1NP	procollagen type 1 *N*-terminal propeptide
p-CA	p-coumaric acid
PCA	protocatechuic acid
PDLSCs	human periodontal ligament stem cells
*PPARα*	peroxisome proliferator-activated receptor alpha
*PPARγ*	peroxisome proliferator-activated receptor gamma gene
PPARγ	peroxisome proliferator-activated receptor gamma
PTH	parathyroid hormone
RANKL	receptor activator of nuclear factor kappa-Β ligand
ROS	oxygen species
*Runx2*	runt-related transcription factor 2 gene
RUNX2	runt-related transcription factor 2
SMI	structure model index
SOD	superoxide dismutase
*Sp7*	Sp7 transcription factor 7 gene (osterix)
*SPARC*	secreted protein acidic and cysteine rich gene
*SPP1*	osteopontin gene
SPP1	osteopontin
STAT3	signal transducer and activator of transcription 3
T2DM	diabetes mellitus type 2
TAG	triacylglycerol
Tb.N	trabecular number
Tb.Sp	trabecular separation
Tb.Th	trabecular thickness
TNF-α	tumor necrosis factor-alpha
*Traf6*	TNF receptor-associated factor-6 gene
TRAP	tartrate-resistant acid phosphatase
*Trap*	tartrate-resistant acid phosphatase gene
TSH	thyroid-stimulating hormone
*UCP1*	uncoupling protein 1 gene
XO	xanthine oxidase
Zn	zinc
